# Programmed cell death and lipid metabolism of macrophages in NAFLD

**DOI:** 10.3389/fimmu.2023.1118449

**Published:** 2023-01-18

**Authors:** Zhun Xiao, Minghao Liu, Fangming Yang, Guangwei Liu, Jiangkai Liu, Wenxia Zhao, Suping Ma, Zhongping Duan

**Affiliations:** ^1^ Department of Digestive Diseases, The First Affiliated Hospital of Henan University of Chinese Medicine, Zhengzhou, China; ^2^ Beijing Institute of Hepatology, Beijing Youan Hospital Capital Medical University, Beijing, China

**Keywords:** non-alcoholic fatty liver disease, inflammation, macrophages, programmed cell death, lipid metabolism

## Abstract

Non-alcoholic fatty liver disease (NAFLD) has now become the leading chronic liver disease worldwide with lifestyle changes. This may lead to NAFLD becoming the leading cause of end-stage liver disease in the future. To date, there are still no effective therapeutic drugs for NAFLD. An in-depth exploration of the pathogenesis of NAFLD can help to provide a basis for new therapeutic agents or strategies. As the most important immune cells of the liver, macrophages play an important role in the occurrence and development of liver inflammation and are expected to become effective targets for NAFLD treatment. Programmed cell death (PCD) of macrophages plays a regulatory role in phenotypic transformation, and there is also a certain connection between different types of PCD. However, how PCD regulates macrophage polarization has still not been systematically elucidated. Based on the role of lipid metabolic reprogramming in macrophage polarization, PCD may alter the phenotype by regulating lipid metabolism. We reviewed the effects of macrophages on inflammation in NAFLD and changes in their lipid metabolism, as well as the relationship between different types of PCD and lipid metabolism in macrophages. Furthermore, interactions between different types of PCD and potential therapeutic agents targeting of macrophages PCD are also explored.

## 1 Introduction

Non-alcoholic fatty liver disease (NAFLD) is currently the most common liver disease, and affects approximately one-third of the world’s population (1). According to the severity and the pathological phase, NAFLD can be divided into non-alcoholic fatty liver (NAFL), non-alcoholic fat hepatitis (NASH), liver fibrosis and cirrhosis. Although NAFL has no clinically significant, there is evidence suggests that approximately 25% of patients with NAFL progress to NASH (2). The presence of NASH promotes the progression of liver pathology and increases the incidence of adverse outcomes compared to NAFL. In recent years, the global proportion of NAFLD-associated hepatocellular carcinoma (HCC) has increased year by year and may gradually become the main cause of HCC (3). Therefore, it is important to find effective therapeutic agents to block the pathological progression of NAFLD, especially NASH. The transition from NAFL to NASH is the result of a complex multifactorial effect, which involves a complex liver cell population (both parenchymal and non-parenchymal cells) as well as pathological signals from visceral fat and intestine. The pathogenesis of NAFLD was considered to be the “two-hit hypothesis” (4). Lipid accumulation in hepatocytes represents the “first hit”, while other factors such as oxidative stress are referred to as the “second hit”. However, recent studies have suggested that NAFLD progression may be influenced by various factors such as environment, metabolism, gut microbiota, and genetic factors (5, 6). The simultaneous changes of insulin resistance, genetic and epigenetic factors, mitochondrial dysfunction, endoplasmic reticulum stress, microbiota, chronic low-grade inflammation, etc. led to the progress of NAFLD, which was named “multiple parallel hits hypothesis” (7).

The main sources of lipid deposition in the liver include adipose tissue lipolysis, hepatic *de novo* lipogenesis (DNL), and diet, with the former accounting for the majority (8). Excess fatty acids are taken up and intracellularly transported in the liver by hepatocytes, macrophages, and other liver cells. When excess free fatty acids (FFAs) exceed the antioxidant capacity of the body, inflammation occurs. These mechanisms have been well summarized in previous reviews (9, 10). Macrophages are an important component of innate immunity. Macrophages in the liver mainly include tissue-resident Kupffer cells (KCs), monocyte-derived macrophages (MoMFs) and subcapsular macrophages discovered in recent years (11). Subcapsular macrophages play a major role in the defense against infectious agents from the abdominal cavity and are not the topic of this article. KCs are the most abundant tissue-resident macrophages in the mammalian body, accounting for 80-90% of all tissue-resident macrophages (12). KCs are mainly localized in the reticuloendothelial system and sense risk factors from the intestine and adipose tissue as well as multiple signals from the liver microenvironment. KCs constitute the hepatic immune homeostasis and alert when the balance is disturbed. When inflammation is induced, numerous monocytes are recruited to the liver (13). This may contribute to the chronic low-grade inflammation in NAFLD. Considering the important impact of macrophages on hepatic inflammation and their ability to process lipids, they play an important role in the pathological progression of NAFLD. Programmed cell death (PCD) of macrophages is closely associated with the development of inflammation (14). This paper mainly focuses on the effects of lipid metabolism and PCD on the phenotype of macrophages in NAFLD and the relationship between them. The possibility of targeting PCD of macrophages in the treatment of NAFLD has also been explored.

## 2 Effect of macrophages on the inflammatory response in NAFLD

**Figure 1:**
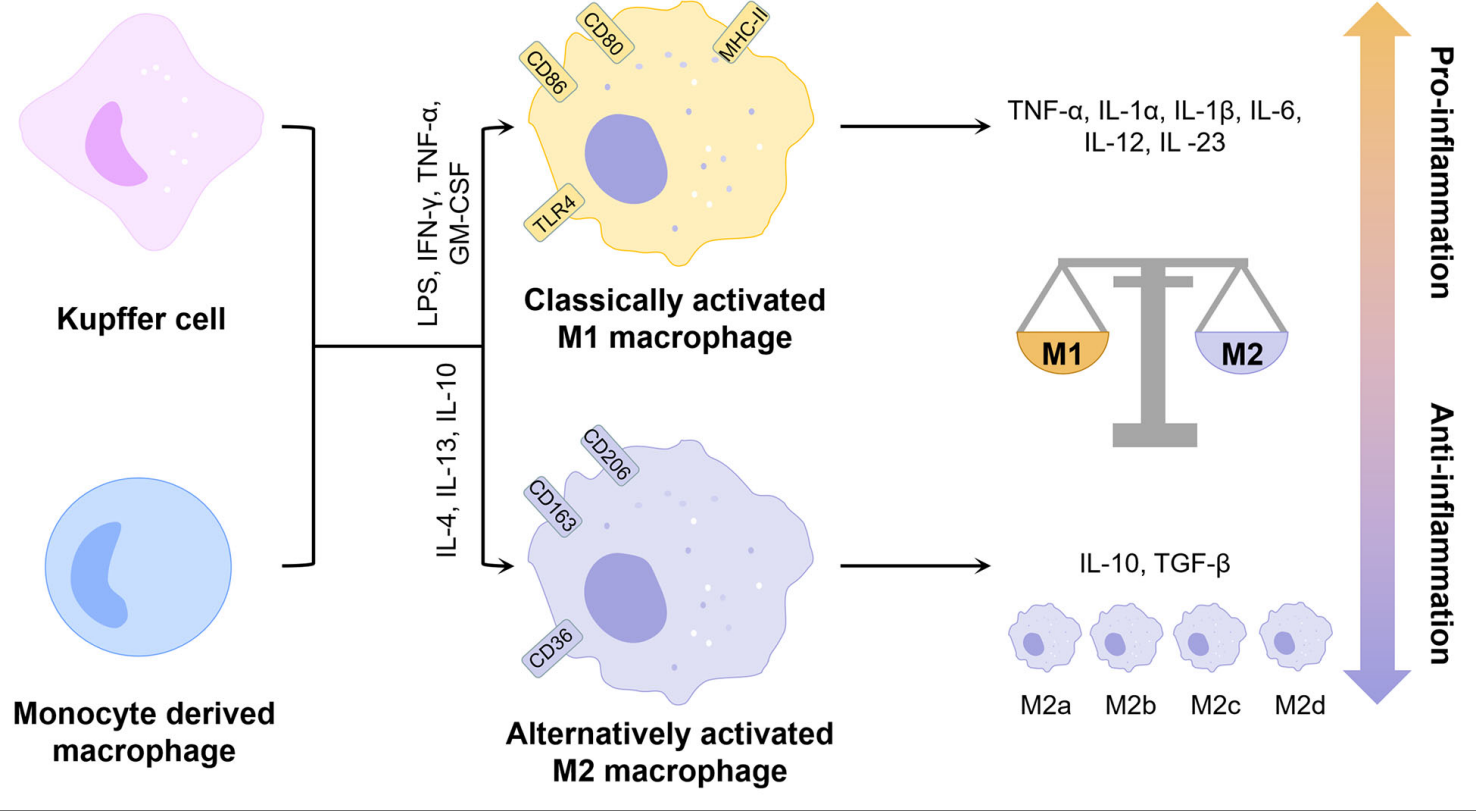
Phenotype and function of liver macrophages. KCs and monocyte-derived macrophages can be polarized by different factors into two distinct phenotypes, classically activated M1 and alternatively activated M2. M2 macrophages can be further classified into multiple phenotypes. Overall, M1 exhibits pro-inflammatory properties and M2 exhibits anti-inflammatory properties. The balance between the two determines the direction of liver inflammation. GM-CSF, granulocyte-macrophage colony-stimulating factor; IFN-γ, interferon-γ; IL, interleukin; LPS, lipopolysaccharide; MHC-II, major histocompatibility complex class IITGF-β, transforming growth factor-β; TLR4, toll-like receptor 4; TNF-α, tumor necrosis factor-α.

Inflammation is a major pathological factor in the progression of NAFLD, and hepatocyte death is one of the crucial triggers of liver inflammation (15). Macrophages play an important role in the inflammatory response in NAFLD due to their ability to clear pathogens and recruit circulating inflammatory cells (2). Typically, macrophages can be classified into two phenotypes, the classically activated M1 type and the alternatively activated M2 type (**Figure 1**). M1 macrophages are induced by lipopolysaccharide (LPS) and Th1 cytokines such as interferon-γ (IFN-γ) and granulocyte-macrophage colony-stimulating factor (GM-CSF) alone or in combination to secrete pro-inflammatory factors such as interleukin 1β (IL-1β), IL-6 and tumor necrosis factor-α (TNF-α), while M2 macrophages are induced by Th2 cytokines such as IL-4 and IL-13 to secrete anti-inflammatory factors such as IL-10 and transforming growth factor-β (TGF-β) (16). The balance of M1 and M2 macrophages is an important determinant of the pathological changes in the liver under inflammatory conditions. However, the M1 and M2 classification cannot describe macrophages accurately due to the heterogeneity and functional diversity of macrophages. More specific markers of different macrophage phenotypes are needed.

KCs are liver-resident macrophages and account for 15% of all liver cells (17). It is now clear that KCs originate from yolk-sac-derived erythro-myeloid progenitors expressing colony stimulating factor 1 receptor (CSF1R) (18). In the context of NAFLD, KCs are the major source of cytokines and chemokines (19). For example, KCs promote steatosis and insulin resistance by secreting IL-1β to downregulate peroxisome proliferative activated receptor α (PPARα) expression in hepatocytes (20). A previous study has demonstrated that consumption of KCs attenuates high fat or high sucrose diet-induced NASH in rats (21). On the one hand, portal vein-derived LPS can bind to toll-like receptor 4 (TLR4) on the surface of KCs to induce their polarization toward M1 pro-inflammatory phenotype and enhance pro-inflammatory cytokines including monocyte chemoattractant protein-1 (MCP1, also known as C-C motif chemokine ligand 2, CCL2), TNF-α and IL-6 expression *via* yes-associated Protein (YAP) (22). On the other hand, KCs can also promote hepatocyte apoptosis and inflammatory progression by producing TNF, TNF-related apoptosis-inducing ligand (TRAIL) and factor associated suicide (Fas) ligand through phagocytosis of apoptotic bodies (23). In addition, lipotoxic hepatocytes can release mitochondrial DNA (mtDNA) to induce nuclear factor kappa-light-chain-enhancer of activated B cells (NF-κB) dependent inflammation by binding to transmembrane protein 173 (TMEM173 or STING) on the surface of KCs (24), while KCs themselves can activate the NOD-like receptor thermal protein domain associated protein 3 (NLRP3) inflammasome *via* mtDNA released from mitochondria thereby promoting the progression of NASH (25).

MoMFs are recruited to the liver under inflammatory conditions and transformed to different phenotypes in response to stimulation by complex cytokines. Recent studies have confirmed that lipotoxic hepatocytes can secrete extracellular vesicles rich in MicroRNA 192-5p (26), C-X-C Motif Chemokine Ligand 10 (CXCL10) (27), ceramide (28) to promote M1 polarization of MoMFs and secrete pro-inflammatory cytokines. Meanwhile, extracellular vesicles can also promote the recruitment of MoMFs through an integrin β1 (ITGβ1)-dependent pathway (29). Except for CXCL10, various chemokines including CCL2 played important roles in the infiltration of MoMFs and M1 polarization, while inhibition or knockdown of CXCL10 and CCL2 showed inhibition of liver macrophage infiltration and improvement of inflammation in the mouse model of NASH (30, 31). Lymphocyte antigen 6C (Ly6C) is a marker of mouse monocytes, which divides circulating monocytes into two major subpopulations, Ly6C^hi^ and Ly6C^lo^ (32). The Ly6C^lo^ subgroup shows anti-inflammatory properties, while the Ly6C^hi^ subgroup exhibits pro-inflammatory properties and constitutes the main pathological mechanism of NASH progression. Ly6C^hi^ MoMFs can be converted to Ly6C^lo^ MoMFs after the clearance of apoptotic hepatocytes (33). A dual C-C Motif Chemokine Receptor 2 (CCR2)/CCR5 antagonist, cenicriviroc (CVC), demonstrated improvement in NASH-related liver fibrosis and inhibition of steatohepatitis progression in a completed phase II clinical trial enrolling 289 patients with NASH (34). A study in a mouse model of NASH showed that CVC inhibited Ly6C^hi^ MoMFs infiltration and fibrosis progression, but had no direct effect on macrophage polarization (35).

Due to the immune tolerance characteristic of the liver, KCs are mainly involved in maintaining inflammatory homeostasis, while MoMFs play a major role in acute and chronic liver inflammation (32). Under homeostatic conditions, the hepatic macrophage population contains only a small amount of MoMFs. KCs could directly inhibit the inflammatory response of MoMFs by secreting miR-690-containing exosomes, whereas miR-690 of KCs showed low expression during the progression of NASH (36), which might promote the recruitment and pro-inflammatory transformation of MoMFs. In addition, activated KCs can promote the recruitment of Ly6C^hi^ MoMFs by secreting CCL2 (31). KCs proliferate for self-renewal in the liver, but the mechanism of renewal is impaired during NASH. When KCs are depleted, some Ly6C^hi^ MoMFs are even able to convert to KCs to replenish the hepatic KCs pool under specific factors (37). However, compared with embryo-derived KCs, monocyte-derived KCs do not effectively promote hepatic triglyceride storage and exhibit pro-inflammatory properties in the liver, thereby exacerbating liver injury in NASH (38).

## 3 Lipid metabolism in macrophages

**Figure 2:**
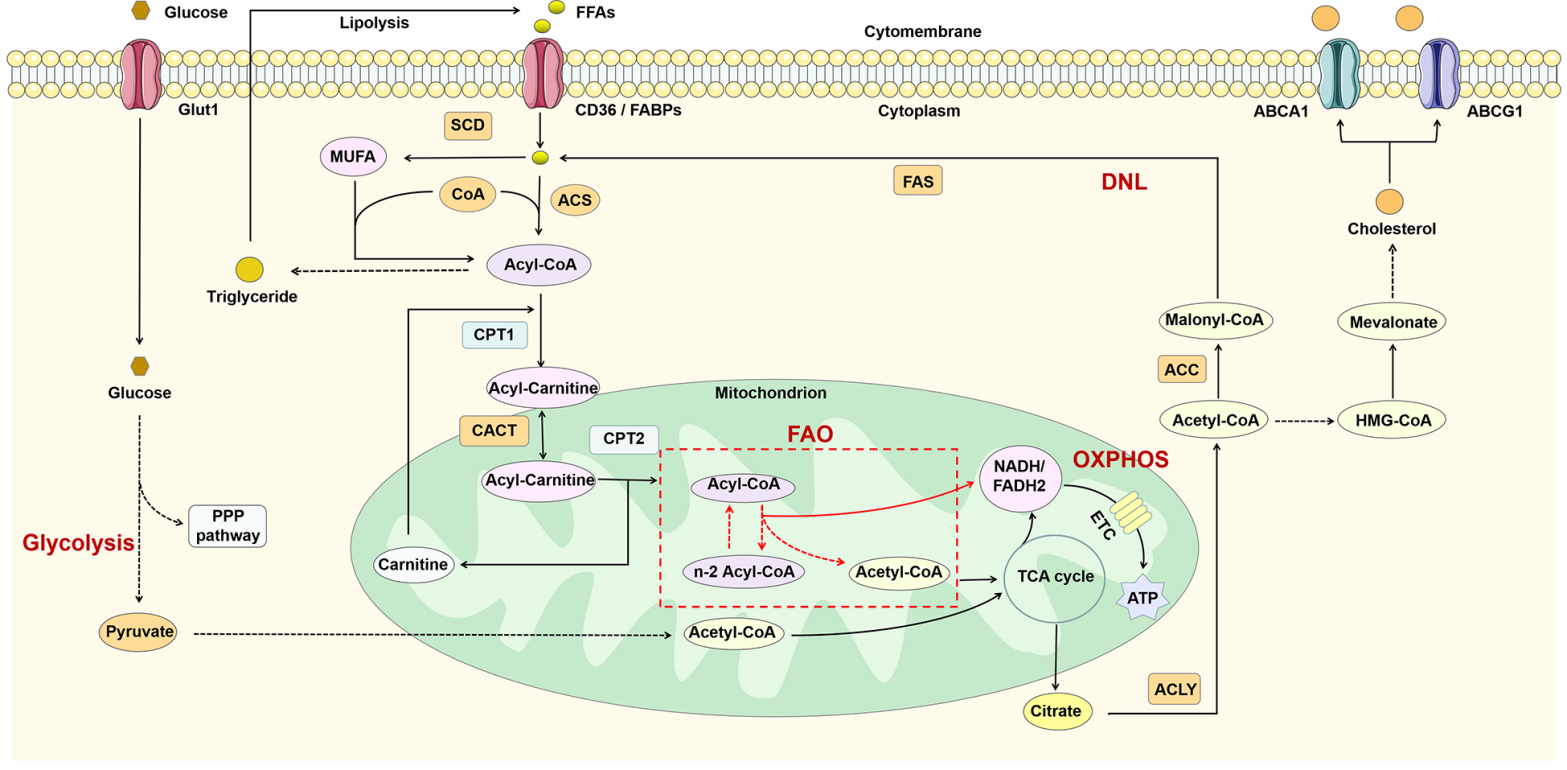
Lipid metabolism in macrophages. FFAs are catabolized to acyl-CoA by the action of ACS and thus participate in triglyceride synthesis and FAO. FAO can participate in the TCA cycle by producing acetyl-CoA and also promote OXPHOS by increasing NADH/FADH2 production. In addition, glucose can also produce acetyl-CoA through glycolysis. The citrate generated by the TCA cycle is reconverted to acetyl-CoA in the cytoplasm by ACL, thus participating in DNL and cholesterol synthesis. ABCA1, ATP-binding cassette sub-family A member 1; ABCG1, ATP-binding cassette sub-family G member 1; ACC, acetyl-CoA carboxylase; ACLY, ATP-citrate lyase; ACS, acyl-CoA synthetase; CACT, carnitine-acylcarnitine translocase; CoA, coenzyme A; CPT1, carnitine palmitoyl transferase 1; DNL, *de novo* lipogenesis; ETC, electron transport chain; FAO, fatty acid oxidation; FAS, fatty acid synthase; FFAs, free fatty acids; Glut1, glucose transporterisoform 1; MUFA, monounsaturated fatty acid; OXPHOS, oxidative phosphorylation; PPP pathway, pentose phosphate pathway; SCD, stearoyl-coenzyme A desaturase; TCA, tricarboxylic acid.

Macrophages require a lot of energy to maintain their function in inflammation. Metabolic reprogramming has an important role in regulating the function of immune cells, and macrophages of different phenotypes usually exhibit different metabolic profiles (39). Macrophages possess lipid-processing function and play an important role in lipid metabolism. Intracellular lipid metabolism involves a series of complex enzymatic reactions (**Figure 2**). FFAs taken up by fatty acid transport proteins (e.g. CD36) and fatty acid binding proteins (FABPs) are transformed to acyl-coenzyme A (acyl-CoA) by the action of acyl-CoA synthase (ACS). Acyl-CoA can not only participate in the synthesis of triglycerides, but also converted to acyl-carnitine by carnitine palmitoyltransferase-1 (CPT1). Subsequently, acyl-carnitine is transported into the mitochondria *via* carnitine-acylcarnitine translocase (CACT) and CPT2 for fatty acid oxidation (FAO) and increases the production of nicotinamide adenine dinucleotide (NADH)/1,5-dihydroflavin adenine dinucleotide (FADH2) thereby promoting oxidative phosphorylation (OXPHOS). The acetyl-CoA produced during this process participates in the TCA cycle and ultimately promotes the production of citrate. Glucose can be converted to pyruvate through glycolysis and then also participate in the TCA cycle through conversion to acetyl-CoA (40). Citrate can be converted back to acetyl-CoA by the action of ATP-citrate lyase (ACLY). Acetyl-CoA is not only involved in *de novo* lipogenesis (DNL), but also in cholesterol synthesis. The excess fatty acids are used for the synthesis of triglycerides and other complex lipids (41), while cholesterol is transported out of the cell under the regulation of ATP-binding cassette sub-family A member 1 (ABCA1) and ATP-binding cassette sub-family G member 1 (ABCG1) (42).

**Figure 3:**
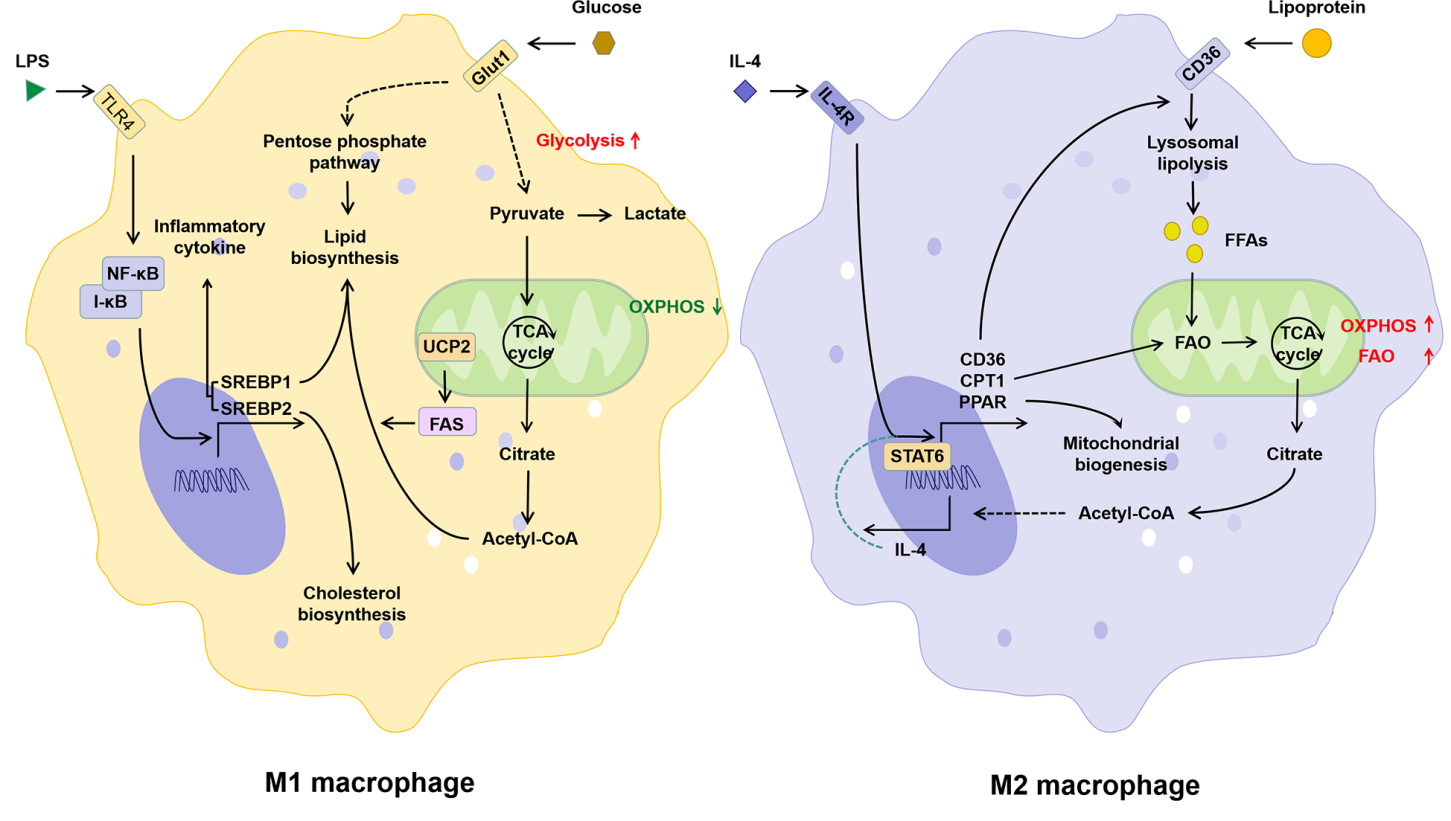
The relationship between lipid metabolism and phenotype of macrophages. Activation of the TLR signaling pathway induced by LPS promotes M1 polarization of macrophages, which is manifested by increased glucose uptake, glycolysis, lipid biosynthesis and decreased OXPHOS. The IL-4 induced M2 polarization of macrophages is manifested as the increase in lipid intake, FAO, and OXPHOS. IL-4, interleukin-4; IL-4R, interleukin-4 receptor; I-κB, inhibitor of NF-κB; LPS, lipopolysaccharide; NF-κB, nuclear factor kappa-light-chain-enhancer of activated B cells; PPAR, peroxisome proliferative activated receptor; SREBP, sterol-regulatory element binding protein; STAT6, signal transducer and activator of transcription 6; TLR4, toll-like receptor 4; UCP2, uncoupling protein 2.

In M1 macrophages, increased glycolysis not only provides ATP more rapidly, but also promotes the TCA cycle and acetyl-CoA production (43). Sterol regulatory element-binding proteins (SREBPs) and fatty acid synthase (FAS), both key regulators of fatty acid synthesis, not only promote lipid biosynthesis in macrophages, but have also been shown to be critical for the induction of M1 polarization of macrophages (44–46). Different from M1, M2 macrophages possess an intact TCA cycle and enhanced mitochondrial OXPHOS, which depends on fatty acid uptake and FAO (43). CPT1 is involved in the production and mitochondrial transport of fatty acid-derived acyl-carnitine. Inhibition of CPT1 blocks mitochondrial FAO and has been shown to inhibit IL-4-mediated M2 polarization of macrophages (47). Thus, classical LPS/IFN-γ-activated pro-inflammatory M1 macrophages *in vitro* exhibit enhanced glucose uptake and anaerobic glycolysis, whereas anti-inflammatory M2 macrophages induced by IL-4/IL-13 exhibit enhanced FAO and OXPHOS (**Figure 3**). Lipid metabolism in M1 macrophages favors lipid synthesis and proinflammatory factor expression, whereas M2 macrophages receive their energy supply through FAO (48, 49). It has been shown that inhibition of FAO inhibits M2 polarization in macrophages (50), while LPS in combination with IFNγ inhibits IL-4-induced M2 repolarization by suppressing OXPHOS in macrophages (51). Due to the high plasticity, macrophages can be phenotypically “repolarized” or “reprogrammed” when induced by the corresponding signals (16). Thus, metabolic regulation may be central to the functional plasticity of macrophages, and FAO plays an essential role in inflammatory and metabolism-mediated phenotypic changes in immune cells.

## 4 Effect of lipid metabolic reprogramming of macrophages on NAFLD

The liver is an important organ of lipid metabolism. Based on the close relationship between macrophage lipid metabolism and inflammation, dysregulated lipid metabolism in NAFLD may promote the progression of inflammation by affecting macrophage function. Modulation of lipid metabolism may have potential therapeutic implications for the progression of inflammation in NAFLD by reshaping the M1/M2 balance. Therefore, it is necessary to further clarify the changes in lipid metabolism of macrophages in NAFLD. KCs express receptors such as MSR1, CD36, and TIM4, which recognize and remove membrane lipid components of apoptotic cells and circulating oxidized low-density lipoproteins (ox-LDL). Subsequently, these lipids are degraded to FFAs and cholesterol by lysosomal acid lipase (LAL), which is involved in HDL synthesis (52). This function of KCs is highly conserved in several species (53), and their expression of genes related to lipid metabolism are more abundant than in other tissue-resident macrophages (54). Given the role of KCs in hepatic immune homeostasis, they may maintain specific functions, particularly tissue-specific functions, dependent on certain metabolites or nutrients (55). Imbalance of lipid homeostasis may lead to pro-inflammatory polarization of KCs thereby inducing inflammation in NAFLD. Improvement of NASH by KCs elimination provides evidence for its driving effect on early NASH (20). CD11c^+^ macrophages may be an important subset driving hepatocyte death-induced inflammation and fibrosis, which promotes disease progression from steatosis to NASH, while KCs are a major source of CD11c^+^ macrophages (56). Macrophage scavenger receptor 1 (MSR1, CD204) mediates lipid uptake and accumulation in KCs and correlates with the degree of steatosis and steatohepatitis in patients with NAFLD. In a fatty acid-rich environment, MSR1 induces a pro-inflammatory response through the JNK signaling pathway, and its blockade inhibits lipid accumulation in KCs, thereby suppressing their pro-inflammatory polarization and the release of cytokines such as TNF-α (57). Similarly, the knockdown of myeloid forkhead box O1 (FoxO1) induced a shift in macrophage polarization from a pro-inflammatory M1 to an anti-inflammatory M2 phenotype and reduced liver macrophage infiltration in a mouse model of high-fat diet-induced NASH (58). At least for now, it seems that lipid overload may promote M1 polarization of KCs and MoMFs through MSR1 and FoxO1, respectively.

Nuclear receptors, including PPARs and liver X receptor (LXR), are ligand-dependent transcription factors and involved in the regulation of lipid and glucose metabolism genes and inflammation-regulated genes (59). This provides another link between macrophage lipid metabolism and inflammation. The three isoforms of PPARs, PPARα, β/δ, and γ, play different but complementary regulatory roles in lipid metabolism and inflammation in the liver (48). The current studies have confirmed the promotion and necessity of PPARγ and PPARβ/δ on M2 polarization of macrophages (60, 61). And LXR can also induce the anti-inflammatory phenotype of MoMFs by inhibiting TLR2, TLR4 and TLR9 and related pathways (62). Interestingly, although LXR was expressed in both hepatocytes and macrophages, an LXRα agonist, DMHCA, selectively activated LXRα in macrophages (63), suggesting the feasibility of targeting LXR in macrophages. In addition, retinoic acid-related orphan nuclear receptor alpha (RORα) is also thought to regulate M2 polarization in macrophages in NAFLD (64). However, the significance of RORα-specific deletion in macrophages for NASH progression remains controversial (65). In addition to regulating lipid metabolism and inflammation, nuclear receptors can also act as redox sensors to sense metabolic stress and thus prevent oxidative damage (66). Due to the important role of oxidative stress in promoting lipid metabolism disorders and inflammation, targeting nuclear receptors may improve NASH progression by regulating macrophage phenotype through antioxidant, anti-inflammatory, and regulating lipid metabolism.

Although the current study confirms the importance of lipid metabolism reprogramming on the pro-inflammatory phenotype of macrophages, changes in the phenotype and lipid metabolism of hepatic macrophage subsets need to be further explored. A recent study showed that depletion of a subset of CD206^hi^ ESAM^+^ KCs or silencing of their fatty acid transporter protein CD36 reversed obesity and steatosis in mice (67). CD36 and CD206 are both commonly considered to be M2 markers (68). These conflicting results further demonstrate the complexity of macrophage function. Furthermore, the role of lipid metabolism in macrophage polarization remains controversial, although it is now generally accepted that glycolysis defines M1 macrophages and FAO defines M2 (43). Glycolysis, OXPHOS and FAO may determine phenotypic shifts through complex interactions rather than a single pathway, which remains to be elucidated.

## 5 Different types of PCD and lipid metabolism in macrophages

**Figure 4:**
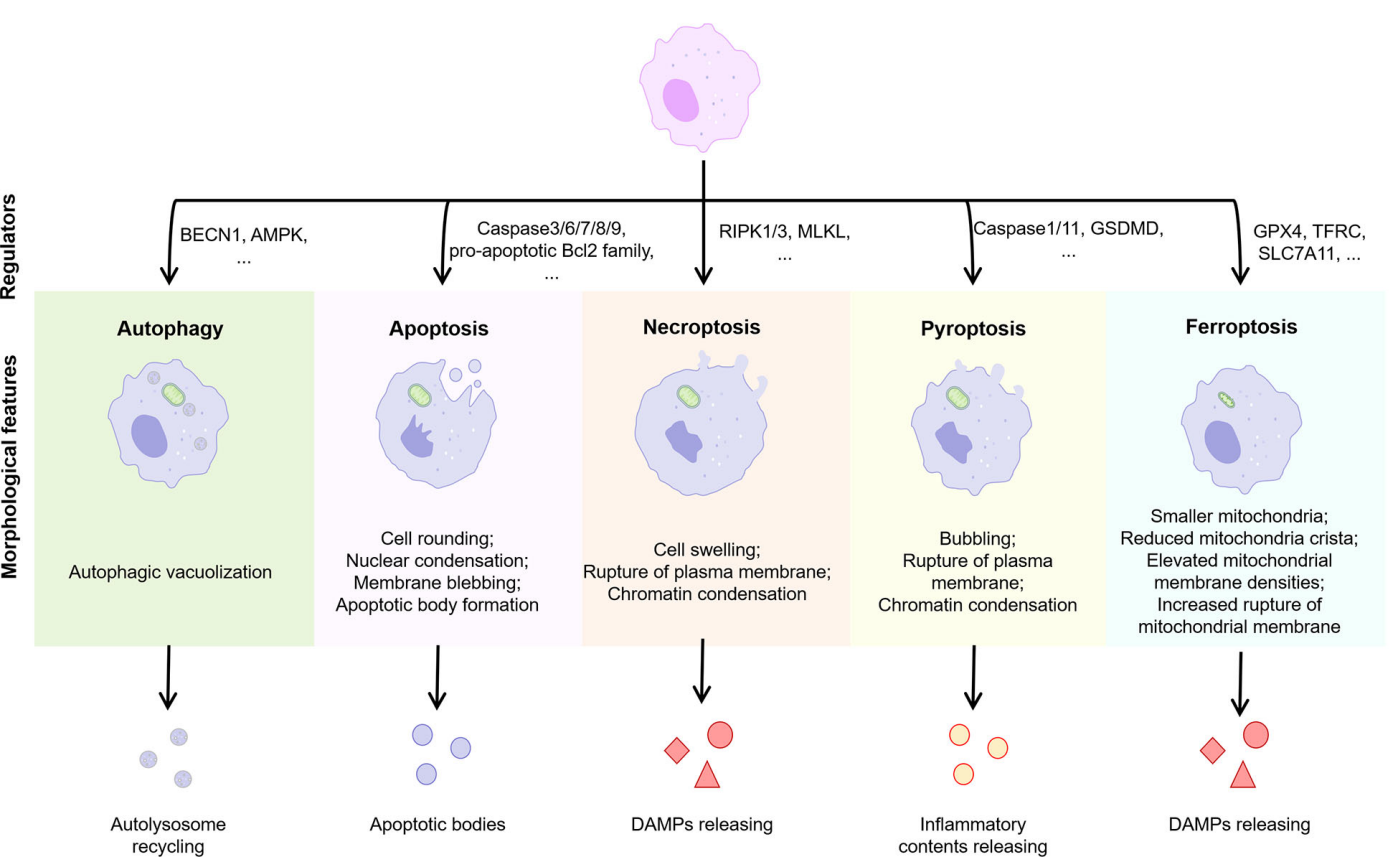
Features of different types of PCD. Normal cells are induced to different forms of death by different stimuli or signals, including autophagy, apoptosis, necroptosis, pyroptosis, and ferroptosis. Different PCD types exhibit different morphological features and play an important regulatory role in the progression of inflammation. BECN1, beclin 1; AMPK, adenosine 5’-monophosphate (AMP)-activated protein kinase; RIPK, receptor-interacting protein kinase; MLKL, mixed lineage kinase domain-like; GSDMD, gasderminD; DAMPs, damage associated molecular patterns; GPX4, glutathione peroxidases 4; TFRC, transferrin receptor; SLC7A11, solute carrier family 7 member 11.

Cells may die from accidental cell death (ACD) or regulatory cell death (RCD). RCD is a strictly regulated form of cell death induced by complex molecular mechanisms, and it is also known as PCD when it occurs in the absence of external environmental interference (69). PCD plays an important role in host defense against pathogens and maintenance of body homeostasis, while its over-activation or tolerance leads to the development of disease. Several types of PCD have been identified including autophagy, apoptosis, necroptosis, pyroptosis, and ferroptosis (**Figure 4**). We focused on these types of PCD in macrophages in the NASH phase of NAFLD.

### 5.1 Autophagy

Autophagy is a catabolic process that degrades damaged organelles and abnormally accumulated proteins *via* lysosomes (70). Autophagy can be classified as macroautophagy, chaperone-mediated autophagy (CMA), and microautophagy according to the pathways of cytoplasmic materials into lysosomes (71). In addition, autophagy can be further classified into selective and non-selective autophagy according to the specificity of degradation substrates. Physiological autophagy is essential for maintaining cellular homeostasis, and its regulation of macrophage function is widely recognized (72). It has been demonstrated that autophagy is impaired in human livers with steatosis or NASH and that promoting autophagy inhibits the pro-inflammatory activation of human macrophage cell lines (73). Typically, autophagy of macrophages in most NAFLD-related studies refers to macroautophagy, the lack of which amplifies hepatic steatosis and/or liver injury through overactivation of the innate immune response. Macrophage-specific deletion of macroautophagy-dependent autophagy-associated proteins such as autophagy protein 5 (ATG5) was shown to increase IL1α and IL1β secretion thereby exacerbating liver inflammation and fibrosis in mice (74). It is suggested that autophagy contributes to the down-regulation of macrophage-induced inflammatory responses, whereas insufficient autophagy may lead to macrophage polarization toward pro-inflammatory M1. Dysbiosis of intestinal flora and increased intestinal permeability in NAFLD patients lead to elevated serum LPS levels, which induce increased secretion of pro-inflammatory factors and reactive oxygen species (ROS) through binding to TLR4, a pattern recognition receptor on the membrane surface of macrophages (75). High concentrations of ROS activated the oxidative stress regulatory switch nuclear factor erythroid 2-related factor 2 (Nrf2) and further induced transcription of antioxidant genes and sequestosome 1 (SQSTM1, also known as p62), thereby inducing p62-dependent selective autophagy in macrophages (76). In contrast, autophagy-deficient hepatic macrophages promoted liver inflammation and fibrosis by enhancing the mitochondrial ROS (mtROS)/NF-κB/IL-1α/β pathway (77).

As a cellular energy sensor, adenosine 5’-monophosphate (AMP)-activated protein kinase (AMPK) can promote macrophage M2 polarization through mitochondrial autophagy (78). However, both free fatty acids and hyperglycemia can lead to autophagy deficiency through the inhibition of AMPK, thereby inhibiting macrophage M2 polarization (79, 80). A study based on liver-specific SQSTM1 knockout mice showed that SQSTM1 induces hepatocyte autophagy by promoting the interaction between AMPK and unc-51 Like Autophagy Activating Kinase 1 (ULK1) and further activates the kelch-1ike ECH- associated protein 1 (Keap1)-Nrf2 signaling pathway to protect the liver from lipotoxicity in mice (81). As an important regulator of nutrient perception, growth, and metabolism, the mammalian target of rapamycin complex (mTORC) phosphorylates ULK1 to inhibit the interaction between AMPK and ULK1 (82), exhibiting a regulatory role in autophagy in contrast to SQSTM1. In addition, AMPK can also inhibit mTORC1 function by phosphorylating Raptor in the mTORC1 complex (83). These studies suggest an antagonism between AMPK and mTORC1 on the regulatory function of autophagy. However, previous studies have demonstrated that SQSTM1 also interacts with Raptor to activate mTORC1 (84). Persistent deficiency of mTORC1 in macrophages has also been shown to inhibit their M2 polarization by inducing lysosomal dysfunction (85). The role of mTORC1 in the regulation of macrophage autophagy and phenotype remains puzzling.

Lipid deposition in the liver impairs local oxygen homeostasis, followed by tissue hypoxia-induced adaptive responses that ultimately affect the homeostasis of hepatic lipid metabolism (86). Since oxygen is transported through the blood, hypoxia means impaired blood microcirculation. Therefore, hypoxia and nutritional disorders occur simultaneously. Nutrient depletion caused by hypoxia activates AMPK and simultaneously inactivates mTORC1 thereby inducing autophagy to maintain cell survival. As a key regulator of hypoxia, Hypoxia-inducible factor 1 subunit alpha (HIF-1α) can broadly regulate the expression of hypoxia-inducible genes and the activation of various signaling pathways (87). A study based on methionine and choline-deficient L-amino acid diet (MCD)-fed mice and NASH patients showed that HIF-1α expression in hepatic macrophages was induced by palmitic acid, thereby reducing autophagic flux and targeting inflammasomes to increase IL-1β production (88). Although HIF-1α and AMPK/mTORC1 regulate macrophage autophagy and phenotypic transformation as important links of hypoxia and energy regulation, respectively, their interactions are still not systematically elucidated.

Currently, studies on HIF-1α and AMPK regulation of lipid metabolism in NAFLD are mainly carried out in hepatocytes (89, 90). The mechanisms by which lipid metabolism reprogramming in macrophages regulates autophagy are still poorly understood. Monoacylglycerol lipase (MAGL), the rate-limiting enzyme in the degradation of monoacylglycerols, not only degrades triacylglycerols to free fatty acids and glycerol, but also metabolizes endogenous cannabinoid receptor ligand 2-arachidonyl acid glycerol to arachidonic acid (91). During chronic liver injury, MAGL inhibited macrophage autophagy to promote inflammation and fibrosis, whereas inhibition of MAGL reduced the number of Ly-6C^hi^ macrophages and increased the number of Ly-6C^lo^ macrophages in an autophagy-dependent manner (92). In high-fat diet-induced obese mice, MAGL deletion also exhibited improved inflammation and insulin resistance in adipose tissue and reduced triglyceride levels in the liver (93). This provides evidence for a link between macrophage autophagy and lipid metabolism reprogramming. In addition, macrophage autophagy reduces programmed death ligand 1 (PD-L1) expression and thus inhibits hepatocellular carcinogenesis, while defective autophagy induces its immunosuppressive phenotype and thus promotes hepatocellular carcinoma progression (94). Given the increased incidence of NAFLD-associated HCC, regulation of macrophage autophagy may be valuable in reducing NAFLD-associated HCC.

### 5.2 Apoptosis

Apoptosis is a Caspase-dependent cell death that includes intrinsic pathways induced by DNA damage, ROS accumulation, and endoplasmic reticulum stress (also known as the mitochondrial pathway) as well as exogenous apoptotic pathways initiated by the binding of other cellular soluble or cell surface ligands including TNF, FasL, or TRAIL to death receptors (95). The Caspase family is a family of evolutionarily conserved cysteine-dependent endonucleases that are primarily involved in cell death and inflammatory responses. Caspases involved in apoptosis are divided into two main categories: initiating Caspases (Caspases-2, 8, 9 and 10) and effector Caspases (Caspases-3, 6 and 7) (96). Current studies have amply demonstrated that Caspases-3, 6, 7, 8, and 9 promote the progression of NASH (97). Among them, Caspases-8 is mainly involved in the exogenous apoptotic pathway, while Caspases-9 is mainly involved in the endogenous apoptotic pathway. Increased positivity of the terminal deoxynucleotidyl transferase-mediated dUTP nick end labeling (TUNEL) assay in liver tissue of NASH patients demonstrates the involvement of apoptosis in the progression of NASH (98). Most of the current studies on apoptosis have been conducted on hepatocytes and few on macrophages. A study based on a high-fat diet-induced mouse model of NAFLD and primary KCs demonstrated that IL-4-activated M2 KCs could release IL-10 to promote apoptosis of M1 KCs thereby reducing liver inflammation and hepatocyte injury in NAFLD (99). This study suggests an intrinsic regulatory mechanism for the balance of M1 and M2 macrophages, i.e., M2 macrophages induce protective apoptosis of M1 macrophages and their dysfunction leads to the accumulation of M1 macrophages in the liver and the progression of inflammation.

Endoplasmic reticulum stress is significantly associated with lipotoxicity and NASH (100). C/EBP homologous protein (CHOP) is a transcription factor downstream of protein kinase RNA-activated-like ER kinase. CHOP is induced by endoplasmic reticulum stress and its deletion prevents apoptosis induced by endoplasmic reticulum stress (101). As endoplasmic reticulum stress can induce TRAIL receptors and activate Caspase-8 (102), this may string endogenous apoptotic and exogenous apoptotic pathways together. Early studies demonstrated that CHOP deficiency promotes macrophage resistance to lipotoxicity and the progression of inflammation in NAFLD (103). Therefore, CHOP may have the effect of inducing apoptosis of M1 macrophages and thus regulating hepatic macrophage homeostasis to protect the liver from steatohepatitis. The role of LXRα in promoting M2 polarization in hepatic macrophages has been discussed above. Paradoxically, a peritoneal macrophage-based study demonstrated that LXRα can inhibit the CHOP pathway induced by endoplasmic reticulum stress and thus inhibit macrophage apoptosis (104). The relationship between CHOP and LXRα in M1 macrophages in the liver obviously needs to be further explored.

The suppressor of cytokine signaling (SOCS) family is a class of proteins with negative feedback regulation on cytokine signaling pathways, including eight members of SOCS1 to SOCS7 and cytokine-inducible SH2-containing protein (CIS) (105). Currently, SOCS1, SOCS2 and SOCS3 are the most studied in macrophages. IL-4-induces SOCS2 expression, IFN-γ induces SOCS3 expression, while SOCS1 can be induced by both (106). Differently, induction of SOCS1 by IL-4 is signal transducer and activator of transcription 6 (STAT6) dependent, whereas induction of SOCS1 by IFN-γ is STAT1 dependent. At the same time, IL-4 induced SOCS1 can inhibit the expression of STAT6 and form a negative feedback signal. A recent study showed that SOCS2 expression in macrophages was negatively correlated with the degree of NASH (107). The study also found that SOCS2 plays a role in inhibiting inflammation and apoptosis *via* NF-κB and inflammasome signaling pathway in macrophages during NASH. In addition, SOCS1 and SOCS3 have been shown to inhibit the exogenous apoptotic pathway in other cell lines such as renal tubular epithelial cells and prostate cancer cells, respectively (108, 109). Overall, SOCS2 may promote M2 polarization and inhibit apoptosis in macrophages, while SOCS1 and SOCS3 may promote M1 polarization and inhibit apoptosis. The three are induced by different signals to inhibit exogenous apoptosis in macrophages of the corresponding phenotype thereby regulating the progression of NASH. Notably, both SOCS1 and SOCS3 have been shown to have inhibitory effects on FAO (110–112). Although these studies were not performed in macrophages, the mechanisms all involved inhibition of the Janus kinase (JAK)/STAT pathway. Different from SOCS1 and SOCS3, the effect of SOCS2 on lipid metabolism has received rarely attention. Although it has been shown to prevent hepatic steatosis due to high-fat diet (113), some studies suggest that it does not promote the increase of FAO in the liver (114). In conclusion, it remains to be clarified whether the SOCS family is involved in the regulation of macrophage polarization and apoptosis by inhibiting FAO through the JAK/STAT pathway.

### 5.3 Necroptosis

Necroptosis is a caspase-independent necrotic cell death program regulated by receptor interacting serine/threonine kinase 1 (RIPK1) and RIPK3. It can be triggered by extracellular stimuli that activate inflammation and cell death. Various innate immune signaling pathways such as TNFR, TLR and interferon receptors (IFNRs) can induce the binding of RIPK1 and RIPK3, which then leads to phosphorylation and translocation of mixed lineage kinase domain-like pseudokinase (MLKL) to the cell membrane, ultimately leading to necroptosis and release of damage-associated molecular patterns (DAMPs) (115). Precisely, RIPK1 induces apoptosis rather than necroptosis when Caspase-8 is present. When Caspase-8 is inhibited, deubiquitinated RIPK1 does not bind to complex 2, but to RIPK3, forming a necrosome complex and subsequently recruiting and activating MLKL (116). Thus, inhibition of Caspase-8 is as important as activation of RIPK3 for necroptosis. In contrast, RIPK1, although involved in the induction of necroptosis, may not be necessary for necroptosis. Depletion of nicotinamide adenine dinucleotide (NAD+) is sufficient to trigger necroptosis in a RIPK3- and MLKL-dependent manner (117), which provides a non-classical necroptotic pathway.

Necroptosis plays an important role in macrophage polarization and inflammation. A study based on aged mice showed that aging led to increased necroptosis in liver macrophages and release of pro-inflammatory factors including TNFα, IL-6 and IL-1β, while necrostatin-1s, a necroptosis inhibitor, significantly reduced M1 macrophages and improved inflammation (118). This study suggests that necroptosis promotes M1 polarization of macrophages. O-acetylglucosamine glycosylation modification (O-GlcNAcylation) is a specific glycosylation modification of intracellular proteins that can affect the localization, function and stability of substrate proteins (119). LPS-activated M1 macrophages exhibit attenuated hexose biosynthetic pathway and protein O-GlcNAcylation, while O-GlcNAc transferase (OGT) mediated O-GlcNAcyclization of RIPK3 prevented the hetero interaction and homo interaction of RIPK3-RIPK1, thereby inhibiting macrophage necroptosis (120). A study based on patients with NASH and choline-deficient L-amino acid-defined diet (CDAA)-induced NAFLD models in mice showed that hepatic RIPK3 correlated with NAFLD severity in humans and mice and RIPK3 deficiency ameliorated CDAA-induced inflammation, fibrosis and carcinogenesis in mice (121). Even though RIPK3 has now emerged as one of the promising targets for the treatment of NASH, the mechanism by which necroptosis regulates macrophage polarization has not been systematically elucidated. Studies based on the RIP3-deficient mouse model demonstrated that RIPK3 promotes the TLR4-NF-κB pathway *via* Rho-associated coiled-coil-containing protein kinase (ROCK)1 and thereby induces M1 polarization of macrophages in the liver (122). The effect of necroptosis on M1 macrophage activation and pro-inflammation may also be paracrine-related. Interestingly, M1 but not M2 macrophages exhibited higher RIPK3 and MLKL expression when BMDMs were intervened with necroptosis inducers (123). In contrast, another study expressed a different result. Blockade of TAK1, the RIPK1 inhibitor, induced more intense necroptosis in M2 but not M1 peripheral blood monocyte-derived macrophages in the context of Caspase inhibition (124). The mechanisms underlying these different results are still unclear. Necroptosis may be involved in both depletion of M2 macrophages and activation of M1 to promote the progression of inflammation in NASH.

There may be a correlation between the necroptosis of macrophages and their lipid metabolism reprogramming. RIPK3 was downregulated in macrophages in HCC and promoted FAO *via* the ROS-Caspase1-PPAR pathway, which induced the reprogramming of fatty acid metabolism and ultimately induced M2 polarization in tumor-associated macrophages (125). An atherosclerosis-based study found that MLKL deficiency exacerbated lipid accumulation despite reducing the occurrence of necroptosis in macrophages (126). Another study based on the high-fat diet-induced NAFLD model in mice also showed that RIPK3 deficiency inhibited inflammation while exacerbating hepatic steatosis (127). These studies suggest that the inhibition of necroptosis may promote increased lipid uptake or *de novo* synthesis of fatty acids. Therefore, the relationship between necroptosis and the reprogramming of lipid metabolism in macrophages remains to be further clarified.

### 5.4 Pyroptosis

Pyroptosis is a form of PCD mediated by inflammasome activation, which is manifested by continuous cellular distension until the cell membrane ruptures, thereby releasing cellular contents to activate an intense inflammatory response. Classical pyroptosis is regulated by the inflammasome composed of NLRP3, apoptosis-associated speck-like protein containing a CARD (ASC), pro-Caspase-1 and gasdermin D (GSDMD) (128). Various exogenous and endogenous signals including LPS and ATP can induce inflammasome formation followed by activation of Caspase-1. Activated Caspase-1 cleaves pro-IL-1β and pro-IL-18 to mature IL-1β and IL-18, while cleaving the pyroptotic substrate GSDMD and forming membrane pores to induce pyroptosis and releasing IL-1β and IL-18 (129). The non-classical pyroptosis pathways are cytoplasmic LPS-mediated activation of Caspase-4/5/11 and cleavage by GSDMD (130). Xu et al. (131) showed that GSDMD and its fragment GSDMD-N protein expression, which induce pyroptosis, were significantly increased in liver tissues of NAFLD/NASH patients and correlated with NAFLD activity score and fibrosis. In contrast, MCD-fed GSDMD^-/-^ mice were free from steatohepatitis and fibrosis, demonstrating the role of GSDMD-mediated pyroptosis in promoting NASH. In addition to GSDMD, most members of the gasdermin family can also induce pyroptosis (132), but their role in NAFLD still needs further evaluation.

Most studies on pyroptosis have focused on hepatocytes, but inflammasomes are mainly expressed in immune cells, especially macrophages (133). As an important regulator of anti-inflammation and antioxidant, Nrf2 is downregulated in the liver of NASH patients (134). Macrophage-specific Nrf2 knockdown promotes ROS and IL-1β production *via* a YAP-NLRP3-dependent manner thereby exacerbating NASH progression (135), suggesting the promotion of macrophage pyroptosis on NASH progression. Meanwhile, the gasdermin family may also induce mtROS release by targeting the mitochondrial membrane, thereby triggering NLRP3 inflammasome activation (136, 137). These studies suggest that pyroptosis may promote NASH progression by inducing ROS release and thus amplifying the cascade of pyroptosis and inflammation. Given the regulation of pyroptosis by the NLRP3 inflammasome, inhibition of the NLRP3 inflammasome may attenuate the inflammatory response of liver tissue by inhibiting macrophage pyroptosis (138). NLRP3 blockade also shows improvement in liver inflammation and fibrosis in atherogenic diet-fed foz/foz mice with NASH (139). In addition to the NLRP3 inflammasome, other inflammasome complexes, such as the NLR family CARD domain containing 4 (NLRC4) inflammasome, can also be involved in NAFLD inflammatory progression by promoting macrophage pyroptosis (140). In conclusion, inflammasome activation induces macrophage pyroptosis on the one hand and mediates macrophage polarization on the other. This provides evidence for a close relationship between liver macrophage pyroptosis and pro-inflammatory polarization in NASH.

As mentioned above, macrophage polarization is regulated by disorders of lipid metabolism. Previous studies have demonstrated that lipids released from dead hepatocytes in NASH activate macrophages to overexpress NLRP3 inflammasome and Caspase-1 (141). Therefore, macrophage pyroptosis may be associated with lipid metabolism. Bile acids are endogenous ligands for nuclear receptors that regulate lipid and energy metabolism (142). As a bile acid receptor, G protein-coupled bile acid receptor 1 (GPBAR1, also known as TGR5)-mediated bile acid signaling plays a key role in integrating glucose, lipid and energy metabolism (143). TGR5 activates PPARα and PPAR-γ coactivator 1 alpha (PGC-1α) to increase mitochondrial oxidative phosphorylation and energy metabolism and inhibit NF-κB-mediated pro-inflammatory cytokine production (144, 145), which is important for the metabolic reprogramming of M2 macrophages. Shi et al. (146) found that TGR5 expression was significantly reduced in the liver tissue of NASH patients and mouse models, while TGR5 knockdown exacerbated liver injury and inflammation and promoted macrophage M1 polarization in mice. Mechanistically, TGR5 signaling inhibits NLRP3-mediated macrophage M1 polarization thereby ameliorating hepatic steatosis and inflammation. Although the available evidence suggests an association between macrophage pyroptosis and lipid metabolism, the regulatory mechanisms are still poorly understood.

### 5.5 Ferroptosis

Ferroptosis is a form of iron-dependent cell death mediated by lipid peroxidation, whose main biochemical features are iron deposition and lipid peroxidation (147). Both increased iron uptake and decreased iron excretion may lead to iron overload, which in turn leads to excessive ROS production and lipid peroxidation through Fenton reaction and enzymatic oxygenation, subsequently triggering ferroptosis. The liver is one of the most important organs for iron storage and metabolism. Due to the abnormal lipid deposition in the liver of NASH patients, this may promote the development of ferroptosis. The correlation between disease progression and liver iron overload in NAFLD patients has been demonstrated (148). A bioinformatics study showed that the grading of liver steatosis was associated with 8 iron death-related genes including *ACSL3*, *ACSL4*, *AKR1C1*, *AKR1C2*, *CS*, *FADS2*, *GSS* and *PGD* (149). Another study also showed that the expression of *SLC11A2*, *CP*, *SLC40A1*, and *ACSL5* was downregulated in the livers of NASH patients compared to healthy livers, while the expression of *FTL*, *FTH1*, *ACSL4*, and *ACSL6* was upregulated (150). Actually, targeted ferroptosis has been shown to improve inflammation in both MCD and choline-deficient, ethionine-supplemented (CDE) diet-induced NASH models in mice (151, 152). Current studies on the role of ferroptosis in NAFLD progression have focused on hepatocytes and HSCs (153). Since liver iron is mainly distributed in hepatocytes and reticuloendothelial system (macrophages), iron deposition in macrophages may play a role in NASH. An earlier multicenter study that included 849 patients with NAFLD has also demonstrated that iron deposition in macrophages is associated with severe NASH and advanced liver histological features (154).

Previous studies have confirmed that iron chelation in M1 macrophages may contribute to the development of chronic inflammation, while iron export from M2 macrophages may promote the growth of adjacent cells in the microenvironment (155), suggesting that macrophage polarization is associated with altered iron metabolism. A study based on BMDMs showed that iron overload increased the levels of M1 products (e.g. IL-6, TNF-α and IL-1β), promoting their polarization to the M1 type while exacerbating steatohepatitis and liver fibrosis (156). This study also showed that iron overload inhibited M2 polarization in BMDMs in the presence of IL-4. This differential performance may be associated with higher expression of Hamp and ferritin heavy chain (FTH)/ferritin light chain (FTL) and lower expression of ferroportin (FPN1, also known as SLC40A1) and iron regulatory proteins 1/2 (IRP1/2) in M1 macrophages compared to M2 (157). Moreover, the stronger antioxidant capacity of M1 macrophages enhanced their resistance to iron overload (158). Thus, under the same conditions, M2 macrophages may be induced to die, while the M1 type survives. As iron may be involved in the regulation of energy production and amino acid catabolism, the regulation of iron metabolism in polarized macrophages may alter the macrophage phenotype. For example, anti-inflammatory M2 macrophages in the tumor microenvironment can be converted to pro-inflammatory M1 macrophages *via* ferroptosis (159, 160). However, whether macrophages have this property in NASH remains to be elucidated. Notably, a study based on human monocytic leukemia THP-1 cell-derived macrophages showed that macrophages exhibit M2 polarization rather than M1 in response to chronic iron overload (161), but this may be related to metabolic changes in tumor-associated macrophages.

There is also a connection between ferroptosis and lipid metabolism, which has been well summarized in a recent review (162). Ferroptosis is characterized by iron-dependent peroxidation with phospholipids containing polyunsaturated fatty acyl (PUFA) chains as substrates. Monounsaturated fatty acids (MUFAs), as inhibitor of iron death (163), can be synthesized *de novo* in cells and participate in membrane lipid composition. Lipid metabolism may control the composition of membrane lipid by regulating the balance of PUFAs and MUFAs.Interestingly, sterol-regulatory element binding protein 1 (SREBP-1), which regulates MUFAs synthesis, is upregulated in M1 macrophages. One possible explanation is that ferroptosis signaling induces M1 polarization in macrophages, while reprogramming of lipid metabolism increases their resistance to ferroptosis. Similarly, not only fatty acid β-oxidation is increased in M2 macrophages, but also lipid transport proteins such as CD36 are upregulated. Given that fatty acid β-oxidation reduces the accumulation of PUFAs and thus inhibits lipid peroxidation (164), and that CD36-mediated lipid uptake increases susceptibility to ferroptosis (165), the ferroptosis-mediated shift in macrophage phenotype may ultimately depend on the disruption of the balance between PUFAs and MUFAs.

## 6 Interaction of signals of different types of PCD in macrophages

**Figure 5:**
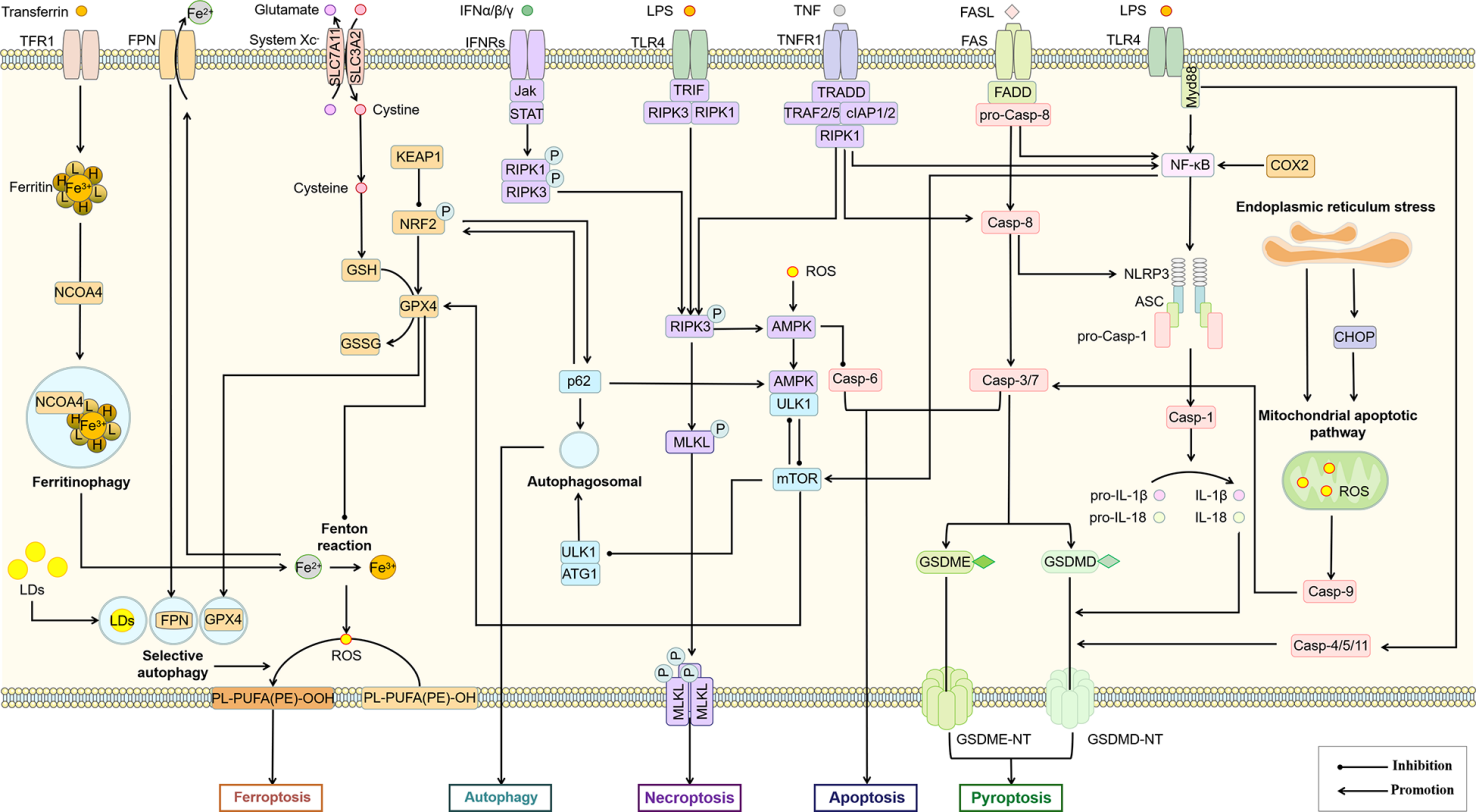
Association of different types of PCD in macrophages. The interaction between different PCD constitutes a complex regulatory network for survival or death of macrophages. AMPK, mTOR, Casp-8, RIPK3, Bcl-2 and p62 may be important nodes in the interaction of autophagy, apoptosis, necroptosis, pyroptosis and ferroptosis in this cell death network. ASC, apoptosis-associated speck-like protein; Casp, Caspase; CHOP, C/EBP homologous protein; cIAP, cellular inhibitor of apoptosis protein; COX-2, cyclooxygenase-2; FADD, Fas-associating protein with a novel death domain; FASL, Fas Ligand; FPN, ferroportin; GSDME, gasdermin E; GSH, glutathione; GSSG, glutathiol; IFN, interferon; Jak, janus kinase; KEAP1, kelch-1ike ECH-associated protein 1; LDs, lipid droplets; mTOR, mammalian target of rapamycin; Myd88, myeloid differentiation primary response 88; NCOA4, nuclear receptor coactivator 4; NLRP3, NOD-like receptor thermal protein domain associated protein 3; NRF2, nuclear factor erythroid 2-related factor 2; ROS, reactive oxygen species; SLC3A2, solute carrier family 3 member 2; TFR1, transferrin receptor 1; TNFR1, tumor necrosis factor receptor 1; TRADD, TNFR1-associated death domain protein; TRAF, TNF-receptor associated factor.

Due to the complex signal environment in the body, there is significant crosstalk between different types of PCD (**Figure 5**). As one of the main inducing pathways of exogenous apoptosis, the TNF signaling pathway also induces necroptosis. The difference is that necroptosis is RIPK3-dependent MLKL activation, while apoptosis is manifested as activation of Caspase-8. A study based on the mouse NASH model induced by MCD showed that Caspase-8 could balance the over-activation of RIPK3-dependent necroptosis, suggesting the mutual inhibition of RIPK3 and Caspase-8 (166). However, the study was performed based on hepatocytes rather than macrophages. Increased autophagy inhibits necroptosis by upregulating ATG16L1 (167) and inhibits apoptosis by inhibiting Caspase-8 activity (168). MLKL, another key regulator of necrotic apoptosis, has been demonstrated to participate in autophagy inhibition in a RIPK3-independent manner in FFC diet (high in fat, fructose and cholesterol) induced NASH mice and palmitic acid treated primary mouse hepatocytes (169). These studies suggest that Caspase-8 is a key node in balancing apoptosis and necroptosis, while MLKL may be an essential node in balancing autophagy and necroptosis. In addition to Caspase-8, Caspase-6 has also been shown to be involved in the interaction between autophagy and apoptosis. As an important participant in autophagy, AMPK can also inhibit apoptosis by phosphorylating Caspase-6 to inhibit its function (170), suggesting an antagonistic mechanism between autophagy and endogenous apoptosis. Notably, the relationship between autophagy and necroptosis is not merely antagonistic. For example, a recent study showed that RIPK3 can directly bind and activate AMPK (171). Considering the mutual inhibition of AMPK and mTOR to regulate autophagic signaling in the downstream, this may be an important link in the balance of autophagy and necroptosis. As an upstream regulator of RIPK3, RIPK1 regulates apoptosis and necroptosis through Caspase-8 and RIPK3, respectively (115). Caspase-8 has been shown to cleave GSDMD to induce pyroptosis (172). These studies all illustrate the complex interaction network among pyroptosis, apoptosis and necroptosis. In experimental and clinical NASH, RIPK1 is phosphorylated and activated mainly in liver macrophages, especially in BMDMs (173). As mentioned above, palmitic acid-induced a decrease in autophagic flux of macrophages (88). However, palmitic acid also induced the activation of RIPK1 (173). Thus, fatty acid-induced inflammatory activation of macrophages was accompanied by an inhibition of autophagy and an increase in apoptosis, necroptosis, and pyroptosis, while the predominant PCD type may be associated with different inducing factors and cytokine expression.

Activation of the NLRP3 inflammasome is not only the initiating link of classical scorch death, but also leads to other types of PCD, including apoptosis, necroptosis, and ferroptosis (132). Prostaglandin-endoperoxide synthase 2 (PTGS2), one of the markers of iron death, regulates the synthesis of cyclooxygenase-2 (COX-2). However, COX-2 increases pro-IL-1β and NLRP3 expression through NF-κB activation and mediates NLRP3 inflammasome activation by enhancing Caspase-1 activation through promoting mitochondrial damage and ROS production (174), which may lead to an increase in pyroptosis. As a major member of the antioxidant system and an important regulator of ferroptosis, glutathione peroxidases 4 (GPX4) also showed the function of inhibiting macrophage pyroptosis (175). Therefore, pyroptosis and ferroptosis may promote each other and thus regulate macrophage pro-inflammatory polarization. The relationship between selective autophagy and ferroptosis has also been widely demonstrated (176). Autophagy promotes ferroptosis through selective degradation of ferritin (177), GPX4 (178), SLC40A1 (179), aryl hydrocarbon receptor nuclear translocator-like (ARNTL) (180), and lipid droplets (181). As a widely recognized inhibitor of autophagy, mTORC1 has also been shown to inhibit ferroptosis by regulating GPX4 synthesis (182). Since DAMPs including proteoglycan decorin (DCN) secreted by ferroptotic cells can bind to advanced glycosylation end-product-specific receptor (AGER) on macrophages and further trigger the production of pro-inflammatory cytokines in an NF-κB-dependent manner. Macrophage-selective autophagy and ferroptosis may promote each other and induce the formation of the NASH inflammatory microenvironment (183). Interestingly, although both pyroptosis and selective autophagy can promote the occurrence of ferroptosis, they do not promote each other. The inhibition of macrophage autophagy has been previously mentioned to be associated with increased pyroptosis. The inhibition of macrophage autophagy has been previously mentioned to be associated with increased pyroptosis. A recent study also confirmed that Tim-4, a phosphatidylserine (PS) receptor, activates liver kinase B1 (LKB1)/AMPKα-mediated autophagy to inhibit NLRP3 inflammasome activation, thereby improving the release of IL-1β and IL-18 from macrophages (184), suggesting that Tim-4^+^ macrophages may inhibit the onset of pyroptosis through autophagy. On the other hand, peritoneal Tim-4 macrophages could inhibit CD8^+^ T proliferation (185), while activated CD8^+^ T cells could release granzyme B to induce increased macrophage pyroptosis and promote NAFLD progression (186). Thus, Tim-4-mediated macrophage autophagy not only directly inhibits pyroptosis, but also indirectly inhibits macrophage pyroptosis by suppressing CD8^+^ T cell activation, suggesting an antagonistic relationship between autophagy and pyroptosis.

It is now generally accepted that the progression of NAFLD is caused by liver lipotoxicity. Possible mediators of lipotoxicity include free cholesterol, saturated free fatty acids, diacylglycerol, lysophosphatidylcholine, sphingolipids, and ceramides (187). Lipotoxic mediators not only induce damage and death of hepatocytes thereby recruiting macrophages, but also directly induce M1 polarization of macrophages. Mitochondria serve as important sites of energy metabolism and regulate liver lipid metabolism and oxidative stress. Changes in mitochondrial metabolism and physiology may underlie the corresponding phenotypes of macrophage activation induced by various signals, including alterations in oxidative metabolism, mitochondrial membrane potential and tricarboxylic acid cycle, as well as the release of mtROS and mtDNA and alterations in mitochondrial ultrastructure (188). Excess ROS attacks biological membranes leading to lipid peroxidation, which not only direct damages phospholipids but also acts as a cell death signal to induce PCD. Numerous studies have confirmed that mitochondrial ROS can induce a variety of PCD in macrophages including pyroptosis (189), autophagy (190), apoptosis (191), necroptosis (192), and ferroptosis (193), suggesting that macrophage polarization and death are closely related to disturbed energy metabolism and oxidative stress. However, it is poorly understood that how dysregulated lipid metabolism in the complex *in vivo* environment leads to different types of PCD in macrophages. Mitochondria play an important role as the energy center in different types of PCD (164), and the development of single cell omics and mitochondriomics may provide valuable information. This aspect is still poorly understood and requires continuous and intensive research.

## 7 Potential drugs targeting PCD of macrophages

As drivers of hepatic steatosis, inflammation, fibrosis and important players in hepatic lipid metabolism, macrophages are attractive therapeutic targets for the treatment of NAFLD. The main strategies currently used to target macrophages include inhibition of monocyte infiltration and inhibition of pro-inflammatory macrophage polarization (194). The improvement of liver inflammation by inhibition of MoMFs infiltration has been well supported by evidence in preclinical studies. A randomized, double-blind, multinational phase 2b study showed that canicriviroc, a dual chemokine receptor CCR2/CCR5 inhibitor, doubled the proportion of patients with at least 1 stage of fibrosis improvement after 1 year despite no improvement in liver inflammation (34). The nuclear receptor family mediates anti-inflammatory polarization of macrophages, thus providing a link between inflammation and lipid metabolism and may be a promising target for NAFLD treatment. Drugs targeting nuclear receptors for the treatment of NAFLD including pan-PPAR agonist (Lanifibranor), PPAR-α/δ agonists (elafibranor), PPAR-α/γ agonist (Saroglitazar) and FXR agonist (Obecholic acid) have been well summarised (48). These drugs may have the effect of modulating both lipid metabolism and phenotypes of macrophages, but more evidence is needed.

PCD is involved in the regulation of the pro-inflammatory polarization of macrophages. Although apoptosis does not induce intense inflammation, apoptosis inhibition has also been considered a therapeutic strategy for NAFLD. As mentioned above, the Caspase family plays an important role in M1 polarization and apoptosis of macrophages. A double-blind, placebo-controlled clinical trial demonstrated that 28 days of treatment with the pan-Caspase inhibitor emricasan significantly reduced ALT and Caspase-3/7 activation in patients with NAFLD (195). However, another clinical study showed that 72 weeks of emricasan treatment did not improve liver histology in patients with NASH fibrosis and may have worsened fibrosis and ballooning (196). Similarly, apoptosis signal-regulated kinase 1 (ASK1) promotes the mitochondrial apoptotic pathway. A multicenter phase 2 clinical trial showed that 24 weeks of treatment with Selonsertib, an ASK1 inhibitor, had no effect on histological inflammation or ballooning despite a reduction in liver fibrosis (197). As the pro-inflammatory properties of hepatocytes and hepatic stellate cells are also regulated by PCD, it is difficult to identify which type or types of liver cells are targeted by these drugs *in vivo*. Multiple types of PCD may act in combination to induce the pro-inflammatory polarization of macrophages. These targeted drugs may have induced other types of PCD and thus failed to improve liver inflammation. Inhibition of Caspases, particularly Caspase-8, may lead to a bias towards necroptosis. Necroptosis of monocytes induced by LPS and pan-Caspase inhibitors increased CXCL1/2, TNF-α and IL-6 expression, whereas inhibition of RIPK3 resulted in a decrease in CXCL1 and CXCL2 and an increase in TNF-α (127). This result suggests that inhibition of a single type of PCD does not resolve inflammation completely. In addition, some natural drugs and their active ingredients have extremely strong anti-inflammation and anti-oxidation capabilities (198), which help regulate the death of macrophages, and are also potential therapeutic drugs for NAFLD. Licochalcone B (LicoB), a main component of the traditional medicinal herb licorice, is a specific inhibitor of the NLRP3 inflammasome which directly binds to never in mitosis A-related kinase 7 (NEK7) and inhibits the interaction between NLRP3 and NEK7 (199). Glycyrrhetinic acid, another active ingredient of licorice, can also improve the damaged autophagy flux and reduce the excessive production of inflammatory cytokines such as TNF-α, IL-6 and IL-1β by regulating the STAT3-HIF-1 pathway of macrophages (200). Curcumin and berberine, two of the most studied natural products for the treatment of NAFLD, have shown positive results in several clinical trials (201, 202). Mechanistic studies have also demonstrated the effect of both on macrophage polarization (203, 204). However, whether regulation of PCD is involved remains unclear.

Given the possible relationship between PCD-regulated macrophage polarization and lipid metabolism, drugs that regulate lipid metabolism may also improve NAFLD by modulating PCD of macrophages and thereby inhibiting the pro-inflammatory polarization. For example, Ezetimibe blocks the NLRP3 inflammasome-IL-1β pathway in macrophages in an autophagy dependent manner, and regulates the interaction between hepatocytes and macrophages through extracellular vesicles (73). In addition, sodium dependent glucose transporters 2 (SGLT2) inhibitors not only control blood glucose by inhibiting the reabsorption of glucose by the proximal tubules of the kidney, but also show regulatory effects on lipid metabolism, such as lipid synthesis and FAO (205). Empagliflozin, one of the SGLT2 inhibitors, also shows the role of regulating the AMPK/mTOR signal pathway to enhance autophagy of liver macrophages in T2DM mouse models with NAFLD (206). The therapeutic strategy of targeting lipid metabolism and PCD for the treatment of NAFLD is gradually being emphasized, and the research progress and clinical trials of inhibitors of the relevant targets are well summarized in a recent review (207). However, it is unclear whether these inhibitors target hepatic macrophages. In **Table 1**, we briefly summarize potential small molecule drugs that may improve NAFLD by regulating PCD of macrophages. The safety and efficacy of these small molecule drugs still need to be supported by more clinical evidence.

**Table 1:** Potential small molecule drugs that regulate macrophage death to improve NAFLD.

Agent	Model	Target/pathway	PCD	Function	Ref
Antcin A	Mouse, high-fat diet; LPS and Nigericin stimulated mouse liver Kupffer cell line	NLRP3	Pyroptosis	Inhibition	(138)
Benzyl isothiocyanate	Mouse, HFCCD diet; LPS with or without cholesterol crystals stimulated primary mouse Kupffer cells,	NLRP3	Pyroptosis	Inhibition	(208)
CpG ODN	T-BHP stimulated RAW264.7 cells	ERK1/2 and Akt signaling pathway	Apoptosis	Inhibition	(209)
Scoparone	Mouse, MCD diet; LPS stimulated RAW264.7 cells	ROS/P38/Nrf2 axis and PI3K/AKT/mTOR pathway	Autophagy	Promotion	(210)
Ezetimibe	Mouse, MCD diet; LPS and palmitate stimulated THP-1 cells	NLRP3 inflammasone-IL1β pathway	Autophagy	Promotion	(73)
Empagliflozin	Mouse, high-fat diet and streptozotocin intraperitoneally injected	AMPK/mTOR pathway	Autophagy	Promotion	(206)
Glycyrrhetinic acid	Mouse, high-fat diet and drinking water containing fructose; Palmitic acid stimulated RAW264.7 and Kupffer cells	STAT3-HIF-1α pathway	Autophagy	Promotion	(200)

MCD, methionine-choline deficient; HFCCD, high-fat diet containing cholesterol and cholic acid; LPS, lipopolysaccharide.

## 8 Discussion

Lipid metabolism disorders are an important factor in the development of NAFLD. Lipid deposition is not the main inducement of cell damage, but it makes cells more vulnerable to the influence of internal and external environments and aggravates cell damage (211). Once the fuse is ignited, disordered lipid metabolism can rapidly exacerbate the hepatic inflammatory cascade. Compared to other liver disease, NAFLD is more likely to be susceptible to severe damage from lipid peroxidation. This may be part of the reason that NAFLD can progress to HCC without the stage of liver cirrhosis. As an important regulator of hepatic inflammatory homeostasis, the M1/M2 imbalance in macrophages leads to the development and progression of inflammation. Based on the important role and huge number of macrophages in liver immune cells, targeting macrophages is of great significance to improve the development and progression of inflammation in NAFLD. Lipids, as key metabolites in macrophage polarization, are closely associated with macrophage function. Conventional opinion suggests that M1 macrophages are dependent on glycolysis for energy while M2 macrophages are dependent on FAO. However, this view has been challenged by some data in recent years, which demonstrate the complexity of macrophage metabolism. Therefore, it remains difficult to answer whether intervention in the lipid metabolic reprogramming of macrophages can improve NASH, and the metabolic profile of different phenotypes of macrophages still needs to be further clarified.

PCD is closely related to the polarization of macrophages. Compared with M2 macrophages, M1 macrophages may be more tolerant to various types of PCD, which leads to its survival in inflammation. At present, most studies targeting macrophages to treat NAFLD only focus on different types of PCD. As mentioned above, various types of PCD crosstalk with each other, which makes it difficult to obtain satisfactory results by blocking a single type of PCD. Other types of PCD can continue to promote the progress of inflammation as a complementary or alternative way. Therefore, elucidating the relationship between different types of PCD and the main regulatory factors will help to effectively regulate the proinflammatory polarization of macrophages. Regulation of macrophage polarization by targeting key regulators of specific macrophage populations to inhibit the pro-inflammatory PCD may be a promising therapeutic strategy for NAFLD. Most studies only provided preliminary evidence for the correlation between PCD and lipid metabolism of macrophages. Based on the regulation of lipid metabolism reprogramming on macrophage polarization, exploring the relationship between PCD and lipid metabolism may help to clarify how PCD regulates the phenotypic transformation of macrophages, and provide a basis for the strategy of targeting macrophages in the treatment of NAFLD.

A suitable animal model is important for mechanistic studies and pre-clinical evaluation of drugs. The pathology of NAFLD is extremely complex. Animal models of NAFLD, whether induced by high-fat, MCD, CDAA diets or specific gene deletions, are only partially reflect the characteristics of NAFLD in humans. This may have led to frustration in clinical trials of numerous drugs that performed well in pre-clinical studies. In addition, the majority of pre-clinical studies were conducted on mice. Species differences lead to inconsistent expression of phenotypic, inflammation, and metabolism-related genes in human and mouse macrophages. The elucidation of the epigenetic and metabolic characteristics of human macrophages is particularly important for the translation from pre-clinical studies to clinical applications. Further exploration of the links between various types of PCD in macrophages and the links between PCD and lipid metabolism may help to identify specific markers of macrophages. This will not only contribute to the development of drugs targeting macrophages for the treatment of NAFLD, but will also be important for the non-invasive diagnosis and assessment of the degree of liver inflammation and disease progression.

## Author contributions

ZX wrote the manuscript. ML involved with project concept. FY, GL, JL and WZ performed data collection. SM and ZD revised the manuscript and were responsible for final approval. All authors contributed to the article and approved the submitted version.
