# Corrigendum: Programmed cell death and lipid metabolism of macrophages in NAFLD

**DOI:** 10.3389/fimmu.2023.1248376

**Published:** 2023-07-10

**Authors:** Zhun Xiao, Minghao Liu, Fangming Yang, Guangwei Liu, Jiangkai Liu, Wenxia Zhao, Suping Ma, Zhongping Duan

**Affiliations:** ^1^ Department of Digestive Diseases, The First Affiliated Hospital of Henan University of Chinese Medicine, Zhengzhou, China; ^2^ Beijing Institute of Hepatology, Beijing Youan Hospital Capital Medical University, Beijing, China

**Keywords:** non-alcoholic fatty liver disease, inflammation, macrophages, programmed cell death, lipid metabolism

In the published article, there was an error in the **Funding** statement. One of the grant numbers for the National Natural Science Foundation of China was displayed as “82205021” when it should be “82205208”. The correct Funding statement appears below.

“This work is supported by National Natural Science Foundation of China (No. 82104772, 81904154, 82205208), China Postdoctoral Science Foundation (No. 2022M721067), The Science and Technology Research Program of Henan Province (No. 222102310502), Key Research and Promotion Project of Henan Province (No. 202102310168), Special Project of Traditional Chinese Medicine Scientific Research of Henan Province (No. 2018JDZX004) and Traditional Chinese Medicine Science Research Project of Henan Province (No. 2022ZY2008).”

The authors apologize for this error and state that this does not change the scientific conclusions of the article in any way. The original article has been updated.

